# The Impact of NaOH on the Micro-Mechanical Properties of the Interface Transition Zone in Low-Carbon Concrete

**DOI:** 10.3390/ma17010258

**Published:** 2024-01-03

**Authors:** Yue Li, Hailong Wang, Lisi Wei, Haolong Guo, Kuo Ma

**Affiliations:** 1 College of Water Conservancy and Civil Engineering, Inner Mongolia Agricultural University, Hohhot 010010, China; ndliyue@163.com (Y.L.); 15754859261@163.com (L.W.); guohl_9610@163.com (H.G.); 2 Inner Mongolia Autonomous Region Water Resources and Hydropower Survey and Design Institute China, Hohhot 010050, China; mk2252329732@163.com

**Keywords:** low-carbon, zeolite powder, glass powder, alkali activation, micro-mechanical property, interfacial transition zone

## Abstract

To tackle carbon emissions from cement production and address the decline in concrete’s mechanical properties due to the substitution of cement with solid waste (glass powder) and natural mineral admixture (zeolite powder) materials, we employed glass powder and zeolite powder to create composite cementitious materials. These materials underwent alkali activation treatment with a 4% NaOH dosage, replacing 50% of cement to produce low-carbon concrete. Nanoindentation tests and mercury intrusion porosimetry (MIP) were employed to uncover the micro-mechanical properties and influencing mechanisms of alkali-activated low-carbon concrete. The results indicate a notable enhancement in the indentation modulus (19.9%) and hardness (25.9%) of alkali-activated low-carbon concrete compared to non-activated concrete. Simultaneously, the interfacial transition zone thickness decreased by 10 µm. The addition of NaOH led to a reduced volume fraction of pores (diameter >100 nm) and an increased fraction of pores (diameter < 100 nm), thereby reducing porosity by 2.6%, optimizing the pore structure of low-carbon concrete. The indentation modulus, hardness and volume fraction of the hydrated phase derived from Gaussian fitting analysis of the nanoindentation statistics showed that NaOH significantly improved the modulus and hardness of the hydration products of low-carbon concrete. This activation resulted in decreased LDC-S-H gel (low-density hydrated calcium silicate Ca_5_Si_6_O_16_(OH)·4H_2_O) and pore content, while the HD C-S-H gel (high-density hydrated calcium silicate Ca_5_Si_6_O_16_(OH)·4H_2_O) and CH (calcium hydroxide crystals Ca(OH)_2_) content increased by 13.91% and 23.46%, respectively. Consequently, NaOH influenced the micro-mechanical properties of low-carbon concrete by generating more high-density hydration products, reducing pore content, enhancing the pore indentation modulus and hardness, and shortening the interfacial transition zone. This study offers novel insights into reducing carbon emissions and promoting the use of solid waste (glass powder) and natural mineral admixture (zeolite powder) materials in concrete, contributing to the advancement of sustainable construction practices.

## 1. Introduction

China has emerged as the world’s leading consumer of concrete, with its cement production holding the top global position. The production of 1 ton of cement clinker results in approximately 0.8 tons of CO_2_ emissions [1]. In 2020, global CO_2_ emissions from cement clinker production reached 1.23 billion tons, contributing to long-term environmental issues such as intensified greenhouse effects and extreme climate events like droughts and floods [2]. Consequently, replacing conventional silicate cement with new materials in the production of low-carbon concrete holds practical significance for ecological sustainability and the achievement of the “peak carbon and carbon neutrality” strategy.

China possesses abundant zeolite resources, and utilizing finely ground zeolite powder (ZP) as a concrete admixture carries substantial economic benefits [3,4]. Previous studies [5,6] indicate that zeolite powder at a dosage of 5–15% significantly enhances the mechanical properties of concrete due to its abundant active SiO_2_ and Al_2_O_3_. These components react with Ca(OH)_2_ generated during cement hydration, producing hydrated calcium silicate–aluminate gel (C-A-S-H) and hydrated calcium silicate gel (C-S-H) to fill pores and improve concrete’s mechanical properties. However, studies by Sun Zheng Ping, Jiang Zheng Wu, and others [7] reveal a decrease in concrete’s mechanical property and workability performance with zeolite powder dosages exceeding 20%. This is attributed to the strong water absorption of zeolite powder, which, under a fixed water–cement ratio, leads to the ineffective dissolution of a large portion of zeolite powder particles. This reduces effective cementitious materials, impacting hydration reactions and diminishing concrete’s mechanical properties. In addition to zeolite, a significant amount of waste glass is annually produced in China, primarily ending up in landfills, consuming land resources and causing environmental pollution [8]. Some researchers grind waste glass into glass powder (GP) to replace cement in concrete production, demonstrating a substantial improvement in mechanical properties [9,10,11]. The high silica content in glass powder promotes a more complete polymerization reaction, partially compensating for performance loss due to reduced cement [12,13]. However, investigations by Petr Hájek [14], Ammari M S [15] and Hasan S [16] show that when glass powder content surpasses 15%, concrete’s mechanical properties are significantly reduced. This is attributed to the lower presence of Ca^2+^ and Al^3+^ in glass powder, leading to reduced contents of hydrated calcium silicate and hydrated calcium silicate–aluminate gel. This results in the formation of more large and connected pores, reducing concrete density and causing a notable strength reduction [17,18], and the use of zeolite powder [19] and glass powder [20] in concrete has lower energy consumption compared to low-carbon materials such as silica fume and fly ash, so zeolite powder and glass powder are selected to prepare low-carbon concrete. However, from the above scholars’ research, it can be seen that the use of the above two powders alone can not meet the conditions for the preparation of low-carbon concrete by replacing cement with large dosage. To enhance material performance, researchers [21] have identified higher hydration activity in cementitious materials in alkaline environments. Alkali-activated cementitious materials offer excellent characteristics, including rapid hardening, high-temperature resistance, and acid corrosion resistance. For pozzolana materials, NaOH is considered the most effective activator [21,22]. Bondar et al. [23,24,25,26] proposed that for low-calcium pozzolana or uncalcined pozzolana containing potassium zeolite, the optimal NaOH dosage is 4%, producing hydration products such as hydrated sodium–alumina–silicate gel (N-A-S-H) to fill pores and enhance concrete’s mechanical properties. For concrete itself [27,28,29,30,31,32], its structural properties depend largely on the nature of the cementitious matrix and the interfacial transition zone between the aggregate and the cementitious matrix; in particular, the interfacial transition zone is considered to be key to the performance of concrete, and because its low cementitious content and porosity is much larger than that of the matrix, this region is considered to be the key to the mechanical properties of concrete.

The aim of this study is to prepare low-carbon concrete using glass powder and zeolite powder, and to investigate the enhancement mechanism of NaOH on the micromechanical properties of low-carbon concrete at the level of the interfacial transition zone. Consequently, this study focuses on preparing composite cementitious materials using different ratios (4:6, 5:5, and 6:4) of zeolite powder to glass powder. These materials undergo alkali activation treatment with a 4% NaOH dosage. Subsequently, activated and non-activated composite cementitious materials replace 50% of the cement to produce low-carbon concrete (LCC). Nanoindentation tests are then employed to investigate the impact of alkali activation on the micro-mechanical properties of LCC. Additionally, mercury intrusion porosimetry (MIP) and fitting methods are utilized to examine the pore structure and hydration phases in the interface transition zone. This comprehensive analysis aims to unveil the influence mechanism of NaOH alkali activation on the micro-mechanical properties of LCC.

## 2. Materials and Methods

### 2.1. Materials

(1) The experimental material chosen for this study is Jidong cement P O42.5; the specific surface area was determined to be 300 m^2^/Kg by the Brønsted method, with an initial setting time of 180 min, a final setting time of 385 min, and demonstrated volume stability meeting the required standards.
(2) The glass powder (GP) used in this study was obtained from Inner Mongolia Hohhot Keman Glass Co., Ltd. (Hohhot, China), appearing as a white powder. Zeolite powder (ZP) was provided by Shijiazhuang Xincheng Mineral Products Co., Ltd. (Shijiazhuang, China), with a gray powder appearance.

**Figure 1:**
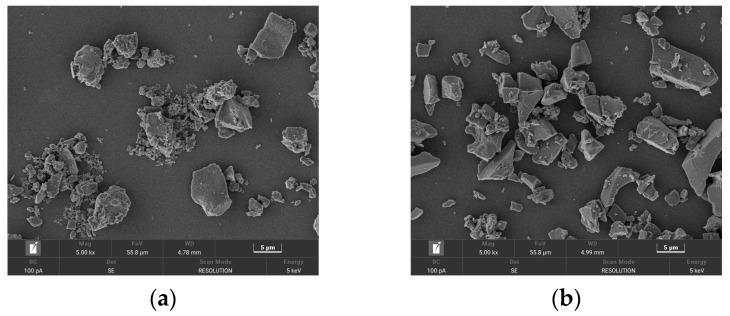
SEM images of (**a**) zeolite powder and (**b**) glass powder. (**a**) ZP; (**b**) GP.

**Figure 2:**
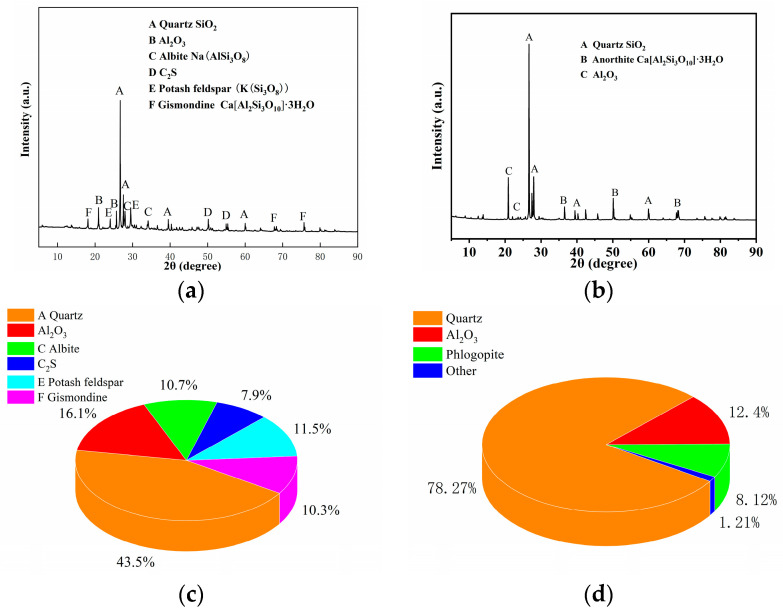
XRD patterns and quantitative phase analysis of (**a**,**c**) zeolite powder and (**b**,**d**) glass powder. (**a**) ZP; (**b**) GP; (**c**) ZP; (**d**) GP.

**Figure 3:**
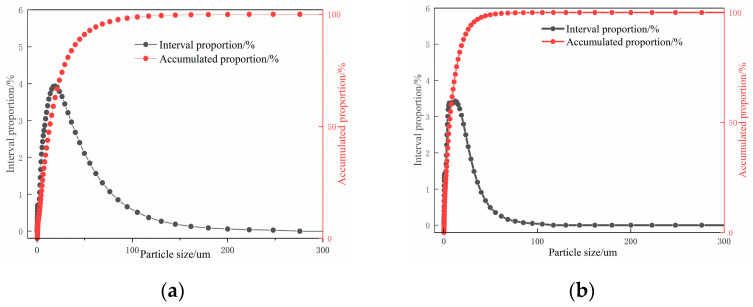
Particle size distribution diagrams of (**a**) ZP and (**b**) GP.

The micromorphology, phase composition, and quantitative analysis of GP and ZP are presented in Figure 1 and Figure 2. Figure 1a,b illustrates that ZP consists of flocculent and irregular block-shaped particles, while GP exhibits various irregular glassy block-shaped and flake-shaped particles. Figure 2a,b indicates that the primary diffraction peaks in ZP correspond to quartz, Al_2_O_3_, sodium feldspar, potassium feldspar, and calcium zeolite crystals, whereas the main diffraction peaks in GP are attributed to quartz and calcium feldspar. The quantitative analysis in Figure 2c,d reveals that quartz constitutes the highest content in GP, at 78.27%, while in ZP, the quartz content is 43.5%, and sodium feldspar crystals constitute 56.5%. Table 1 provides the chemical compositions of ZP and GP. Particle size distribution, obtained using a Horiba-LA-920 laser particle size analyzer, is depicted in Figure 3a,b, indicating a median particle size of 13.32 µm and an apparent density of 590.41 m^2^/kg for ZP, and a median particle size of 5.97 µm and an apparent density of 1060.92 m^2^/kg for GP.

(3) Fine aggregate: ordinary river sand; (4) Coarse aggregate: 5–25 mm crushed stone with continuous grading, bulk density 1550 kg/m^3^, and apparent density 2680 kg/m^3^; (5) Water: ordinary tap water; (6) Admixture: Polycarboxylate water reducer from Jiangsu Zhaojia Building Materials Technology Co., Ltd. (Suzhou, China), presented as a white powder with a water-reducing rate of 20% and a moisture content of 2.3%. (7) Activator: pure NaOH powder.

### 2.2. Mix Ratio Design

Following the “Standard Performance Test Methods of Ordinary Concrete Mixtures” (GB/T50080-2016) [33], C40 ordinary concrete (OC) was formulated. To produce low-carbon concrete, 50% of the cement was replaced with composite cementitious materials derived from zeolite powder and glass powder. Three different ratios of zeolite powder to glass powder (4:6, 5:5, and 6:4) were employed. The resulting low-carbon concrete groups are designated LCC-A-B and WLCC-A-B, where A represents the zeolite powder content, and B represents the glass powder content. LCC-A-B denotes that the composite cementitious materials did not undergo alkali activation treatment, while WLCC-A-B signifies that the composite cementitious materials underwent alkali activation treatments. Table 2 outlines the mixture ratios for low-carbon concrete.

(LCC-A-B represents the mixing ratio of glass powder to zeolite powder as A:B when it is not activated. WLCC-A-B represents the mixing ratio of glass powder to zeolite powder as A:B when it is activated.)

### 2.3. Sample Preparation

After blending zeolite powder and glass powder, both before and after alkali activation, with cement, river sand, coarse aggregate, water, and admixtures, the resulting mixture was poured into a mixing bowl to create cubic samples measuring 100 × 100 × 100 mm. After a 24 h period, the samples were demolded and transferred to a curing box for standard curing. Following 28 days of curing, the concrete samples were placed on a cutting machine and sliced into smaller samples with dimensions of 60 mm × 60 mm and a thickness less than 9 mm, including aggregates. These samples were then subjected to grinding using 400, 800, 1200, and 2000-grit silicon carbide sandpaper, followed by a polishing process involving alcohol-based diamond suspensions with particle sizes of 3 µm, 1 µm, 0.5 µm, and 0.25 µm in sequence. The average roughness of the surface of the sample was measured by AFM, which yielded a roughness less than 1/3 of the depth of the indentation. Upon sample preparation, nanoindentation tests and mercury intrusion tests were conducted to analyze the impact of the NaOH activator on the micro-mechanical properties of the interface transition zone in the low-carbon concrete.

### 2.4. Micro-Mechanical Property Test

#### 2.4.1. AFM Atomic Force Microscope Test

Firstly, the specimens were soaked in anhydrous ethanol for 48 h to terminate their hydration; then, the samples with terminated hydration were dried, and the samples were cold-set with epoxy resin. After the epoxy resin was cured, the samples were put into anhydrous ethanol for ultrasonic cleaning for 5 min; then the samples were analyzed by using an AFM of the model of (Bruker Dimension Icon, Karlsruhe, Germany) for the roughness analysis. The samples were then analyzed for roughness using an atomic force microscope (Bruker Dimension Icon, Germany).

#### 2.4.2. Nanoindentation Test Method

**Figure 4:**
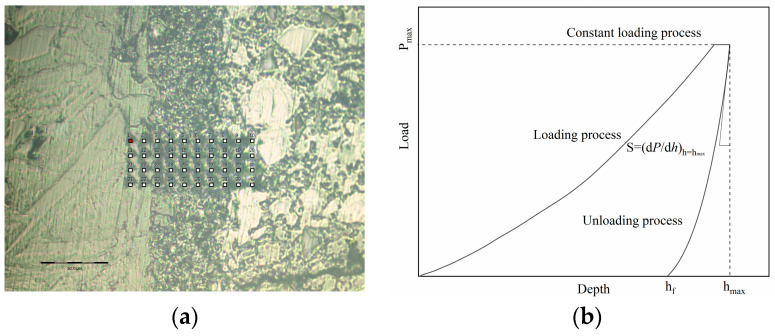
Indentation matrix and load displacement curve of low-carbon concrete samples. (**a**) Indentation matrix; (**b**) load displacement curve.

The nanoindentation tests were conducted using the Hysitron TI 950 UNHT instrument from Austria. The instrument had a maximum load capacity of 1000 mN with a load resolution of 6 nN. The indenter exhibited a total displacement and maximum indentation depth of 1.5 mm and 320 µm, respectively. Its displacement range and accuracy were 80 µm and 0.04, respectively, with a displacement resolution of less than 0.01 nm. The sample stage’s *x* and *y* axes had a resolution of 1.5 µm, and data collection occurred at a frequency of 100 kHz. The interface transition zone between natural aggregates and mortar is commonly acknowledged as a weak area in concrete, exerting a significant influence on concrete’s mechanical properties. To examine variations in the mechanical performance of the concrete interface transition zone pre- and post activation, nanoindentation tests were conducted on each concrete sample group. Initially, suitable interface transition zones were identified for each concrete sample group using an optical microscope. Nanoindentation tests were then executed both horizontally and vertically at 10 µm intervals, commencing 20 µm from the aggregate edge. This process yielded a 10 × 4 grid of indentation matrices with a matrix area of 90 × 30 µm. As shown in Figure 4a, three indentation matrices were selected on the surface of each concrete sample for nanoindentation testing of the interfacial transition zone and resulted in indentation curves as shown in Figure 4b. Following this, a load-controlled mode was employed, with the indenter being linearly loaded to 2 mN at a rate of 0.2 mNs^−1^. After maintaining the load for 10 s, the indenter underwent linear unloading at a rate of 0.2 mNs^−1^. Throughout the testing process, the recorded values of load and displacement within the tested sample were used to construct the load–displacement curve (p–h curve), as depicted in Figure 4b.

#### 2.4.3. Mercury Intrusion Porosimeter (MIP) Method

A Quantachrome Poremaster GT-60 Instrument was utilized for pore structure analysis employing the mercury intrusion porosimeter (MIP) method. As MIP solely measures connected pores, an additional non-destructive testing method of ultrasonic pulse velocity (UPV) was conducted to accurately assess the durability of cement. The pressure range was set at 0.02–33,000 Pisa to determine the porosity and pore size distribution of the concrete.

## 3. Results and Analysis

### 3.1. Roughness Analysis of Concrete ITZ Smaple

**Figure 5:**
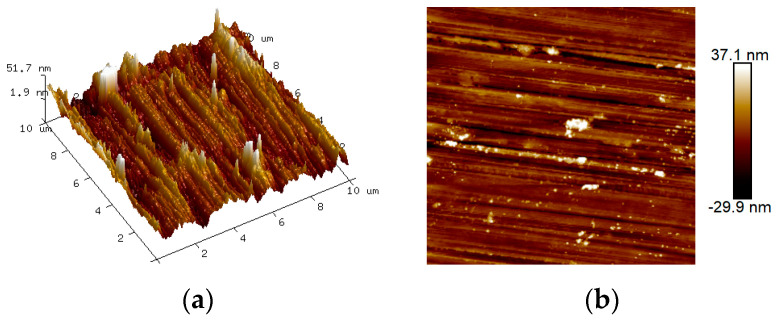
Apparent morphology of each group of concrete; (**a**) 3D apparent morphology; (**b**) 2D apparent morphology.

The flatness of the sample surface is most important for the repeatability of the nanoindentation test data, so before analyzing the micromechanical properties (indentation modulus and hardness), the roughness of the polished samples was first analyzed. It has been shown [34] that the apparent morphology of the ITZ region can be analyzed by AFM testing, and the roughness can be obtained. Roughness is considered when the average roughness is less than 1/3 of the depth of indentation in a nano-indentation test. The interference caused by roughness was eliminated, as shown in Figure 5. Then, the average roughness values of the ITZ region of each group of samples were obtained, as shown in Table 3. The average roughness values of the surface of each group of concrete samples were lower than 1/3 of the maximum indentation depth, which was sufficient to eliminate the influence of roughness on the nanoindentation test data. According to the relevant studies [35], mean square roughness can succinctly control the difference in indentation depth, and it is widely used and recognized.

### 3.2. Nanoindentation Modulus and Hardness Analysis

**Figure 6:**
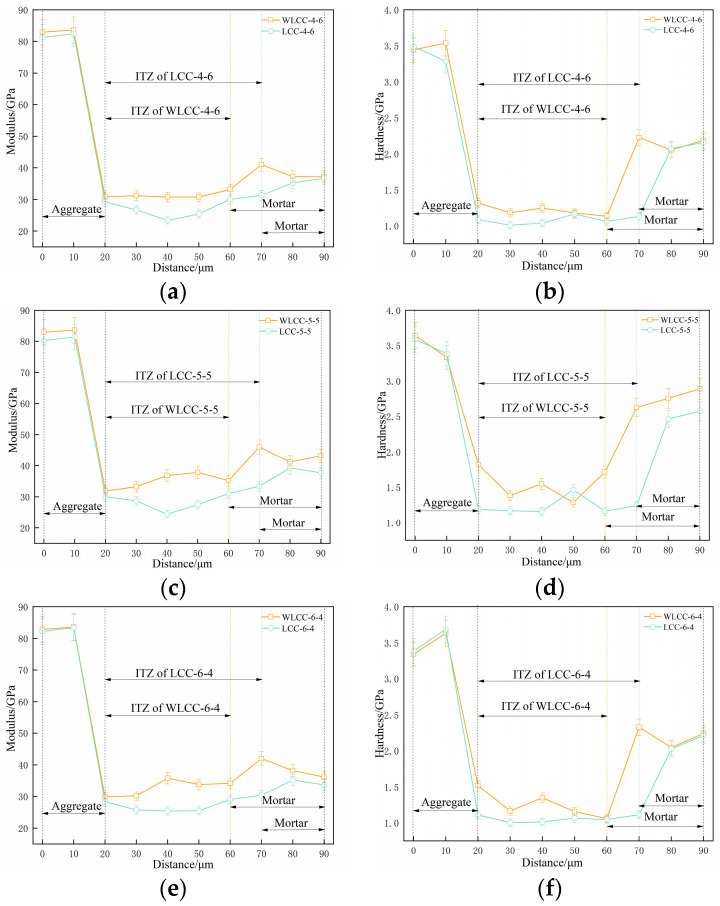
Indentation modulus and indentation hardness of each group. (**a**) Modulus/GPa; (**b**) hardness/GPa; (**c**) modulus/GPa; (**d**) hardness/GPa; (**e**) modulus/GPa; (**f**) Hardness/GPa.

Nanoindentation testing has proved to be a valuable method for assessing the mechanical properties of aggregates, interface transition zones, and mortar in concrete. The obtained results offer explicit insights into the microstructural characteristics of concrete materials. Furthermore, the nanoindentation modulus and hardness of aggregates, interface transition zones, and mortar matrices can be computed, enabling a direct comparison of micro-mechanical properties in the interface transition zone among various concrete samples before and after activation [36,37,38]. From Figure 6a–f, it can be seen that the indentation modulus and hardness of the ITZ and mortar of the low-carbon concrete WLCC-4-6, WLCC-5-5 and WLCC-6-4 after the excitation compared to the low-carbon concrete LCC-4-6, LCC-5-5 and LCC-6-4 before the excitation underwent a large increase, while the indentation modulus and hardness of the WLCC-4-6, WLCC-5-5 and WLCC-6-4 groups were reduced by 10 μm compared to LCC-4-6, LCC-5-5 and LCC-6-4. The WLCC-6-4 group of low-carbon concrete interfacial transition zone thicknesses were reduced by 10 μm compared to LCC-4-6, LCC-5-5 and LCC-6-4. Among the components, aggregates display the highest nanoindentation modulus and hardness, followed by the mortar matrix, while the interface transition zone consistently exhibits the lowest values. Evidently, the interface transition zone emerges as the weakest segment of concrete. It is crucial to highlight that post-activation, there is a significant increase in the nanoindentation modulus and hardness of low-carbon concrete. This phenomenon is likely attributed to the OH^−^ ions inducing substantial SiO_2_ dissolution in low-carbon concrete, resulting in the generation of silicate ions and the transformation of the low-density hydration phase into a high-density counterpart. This transformation contributes to a notable augmentation in the modulus and hardness of the hydration phase, while concurrently reducing porosity content. Consequently, there is an enhancement in the micro-mechanical properties of the interface transition zone.

**Figure 7:**
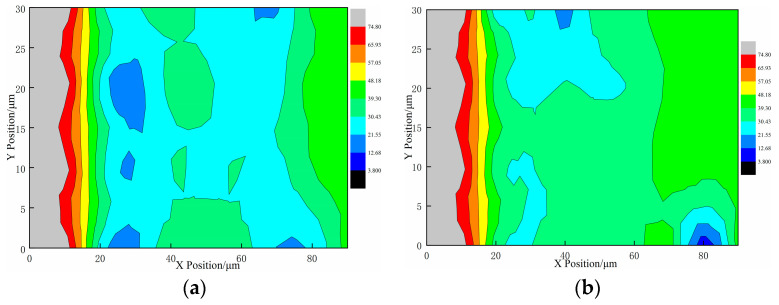
Analysis of elastic modulus contours for (**a**) WLCC-5-5 and (**b**) LCC-5-5. (**a**) Contour analysis diagram of LCC-5-5; (**b**) contour analysis diagram of WLCC-5-5.

Examining the average values of nanoindentation hardness and modulus outlined in Table 4, it is apparent that WLCC-5-5 exhibits the most noticeable enhancement in both nanoindentation modulus and hardness within the mortar and interface transition zone. These values closely align with those of ordinary concrete (OC) in comparison to the other groups. In contrast to LCC-5-5, WLCC-5-5 demonstrates a 19.9% increase in nanoindentation modulus and a 25.9% increase in nanoindentation hardness specifically within the interface transition zone. For a more intuitive visualization, the elastic modulus contour maps in Figure 7a,b offer additional insights. As can be seen from Figure 7, the addition of NaOH results in a reduction of the low-density phase within the interface transition zone, and an observable indentation phenomenon is detected. This leads to a 10 µm reduction in thickness compared to LCC-5-5. To delve deeper into the influence of NaOH on the microstructural characteristics of the interface transition zone in low-carbon concrete, a comparative analysis of porosity and hydration phases between WLCC-5-5 and LCC-5-5 is undertaken.

### 3.3. MIP Analysis

**Figure 8:**
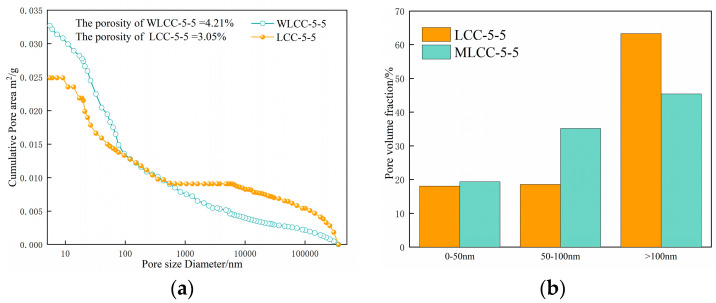
Accumulated pore volumes and volume fractions of (**a**) WLCC-5-5 and (**b**) LCC-5-5. (**a**) Contour analysis diagram; (**b**) pore volume fraction.

Through nanoindentation testing, the analysis of micro-mechanical properties reveals a notable enhancement in the micro-mechanical property of low-carbon concrete with the inclusion of NaOH. Subsequently, the MIP test is conducted to scrutinize the pore structure of WLCC-5-5 and LCC-5-5. As depicted in Figure 8a,b, WLCC-5-5 exhibits a higher volume fraction of pores smaller than 100 nm (referred to as harmless pores in this study) and a lower volume fraction of pores within the 100 nm range (referred to as harmful pores in this study) compared to LCC-5-5. Prior research has established that pores larger than 100 nm adversely affect the micro-mechanical property of concrete, whereas pores smaller than 100 nm have negligible impact. Also given in Figure 8a is the overall porosity of WLCC-5-5 compared to LCC-5-5. The overall porosity of WLCC-5-5 is reduced by 1.16% compared to that of LCC-5-5. Thus, it can be deduced that the addition of NaOH reduces harmful pores and increases harmless pores in the interface transition zone of low-carbon concrete, thereby enhancing the compactness of the interface transition zone. This leads to an indentation phenomenon, a reduction in the thickness of the interface transition zone, and an improved micro-mechanical property. This is consistent with the conclusions drawn from the study of [39] on porosity, where an increase in the volume fraction of pores above 100 nm and a decrease in the overall porosity and volume fraction of pores above 100 nm represent an increase in the compactness of the interfacial transition zone, which leads to a decrease in the thickness of the interfacial transition zone, and thus improves the micro-mechanical properties of the ITZ.

### 3.4. Hydration Phase Analysis Based on Nanoindentation Test

**Figure 9:**
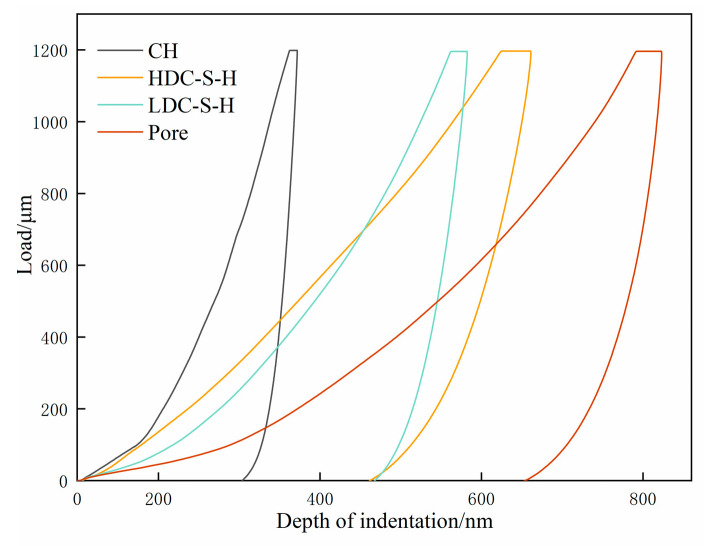
Typical p–h curves of different phases [43].

The MIP results outlined earlier lead to the conclusion that the micro-pore structure of low-carbon concrete can be optimized by introducing NaOH, thereby enhancing its micro-mechanical properties. This phenomenon, observed in prior studies, is linked to variations in the internal hydration degree of concrete and the structure of hydration products [40]. To explore the disparities in hydration phases between LCC-5-5 and WLCC-5-5, Gaussian [41] fitting was applied to the nanoindentation statistical data of both, yielding peak values (P) for nanoindentation modulus and hardness in mortar and different phases. The changes in hydration phases of low-carbon concrete before and after activation were analyzed. According to previous studies [42], the elastic modulus and hardness values of various phases in cement-based materials are different. Specifically, the elastic modulus falls within the ranges of 0 to 13, 13 to 26, 26 to 40, and 40 to 55 GPa for capillary pores, low-density calcium silicate hydrate gel (LDC-S-H), high-density calcium silicate hydrate gel (HDC-S-H), and calcium hydroxide (CH), respectively. For simplicity, only these four phases are considered, while phases with an elastic modulus exceeding 70 GPa are disregarded. The corresponding p–h curves for these hydration phases are depicted in Figure 9.

**Figure 10:**
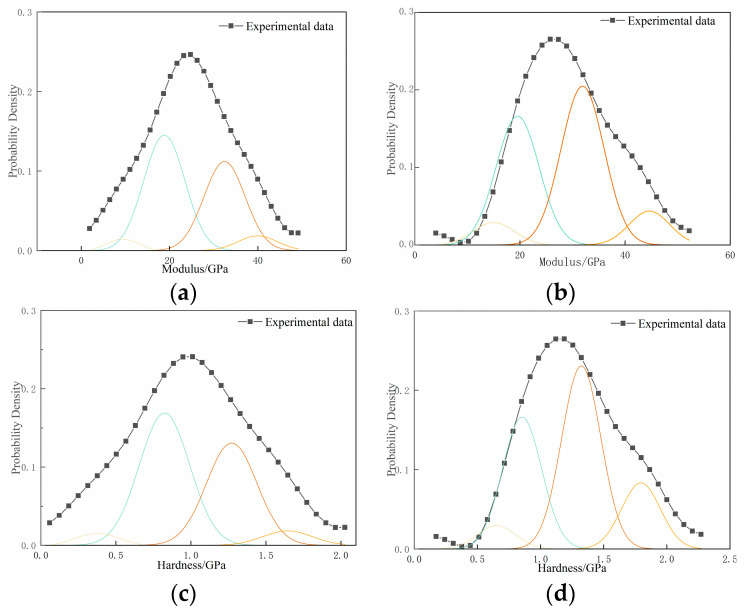
Probability distribution functions of hydration products in WLCC-5-5 and LCC-5-5 phases. (**a**) Modulus of hydrate phase in LCC-5-5; (**b**) modulus of hydrate phase in WLCC-5-5; (**c**) hardness of hydrate phase in LCC-5-5; (**d**) hardness of hydrate phase in WLCC-5-5.

**Figure 11:**
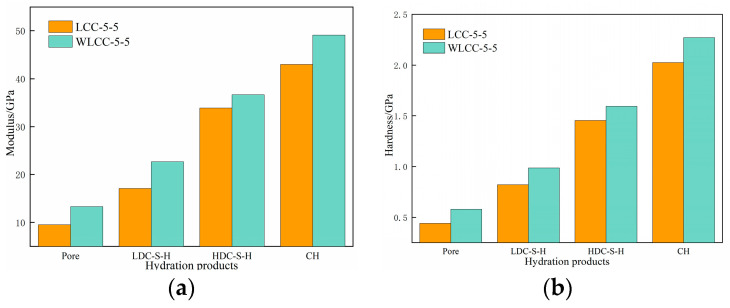
Probability distribution functions of hydration products in (**a**) WLCC-5-5 and (**b**) LCC-5-5 phases. (**a**) Graph of peak elastic modulus of each hydrate phase; (**b**) graph of peak elastic hardness of each hydrate phase.

The nanoindentation modulus and hardness peak values (P) for mortar, as well as the different phases of LCC-5-5 and WLCC-5-5 before and after activation, can be determined through Gaussian fitting, as illustrated in Figure 10 and Figure 11. In Figure 10a–d, the probability distributions of nanoindentation modulus and hardness for WLCC-5-5 and LCC-5-5 samples exhibit similar trends. Four distinct peaks are evident, representing capillary pores, LDC-S-H gel, HDC-S-H gel, and CH, respectively. Applying the Delesse principle [44,45], the specific surface area fraction of concrete is equivalent to its volume fraction, allowing the calculation of the area fraction for each hydration phase to replace the volume fraction. Table 5 presents the fitting results for the average value, standard deviation, and volume fraction of each hydration phase. It is noteworthy that WLCC-5-5 demonstrates superior micro-mechanical properties compared to LCC-5-5. In comparison with LCC-5-5, the indentation modulus and hardness of each phase in the WLCC-5-5 sample are roughly increased by 2 GPa and 0.3 GPa, respectively. As depicted in Figure 11, it is evident that after activation, the nanoindentation modulus of capillary pores increases by 30.23%, and the hardness increases by 43.55%. The nanoindentation modulus of LDC-S-H increases by 20.13%, and its hardness increases by 19.68%. The nanoindentation modulus of HDC-S-H increases by 18%, and its hardness increases by 17.79%. Consequently, it can be inferred that under NaOH conditions, low-carbon concrete can generate more high-density beneficial gel, effectively filling the pores and defects within the concrete. This leads to a dense internal structure and rapid development of micro-mechanical properties in low-carbon concrete.

**Figure 12:**
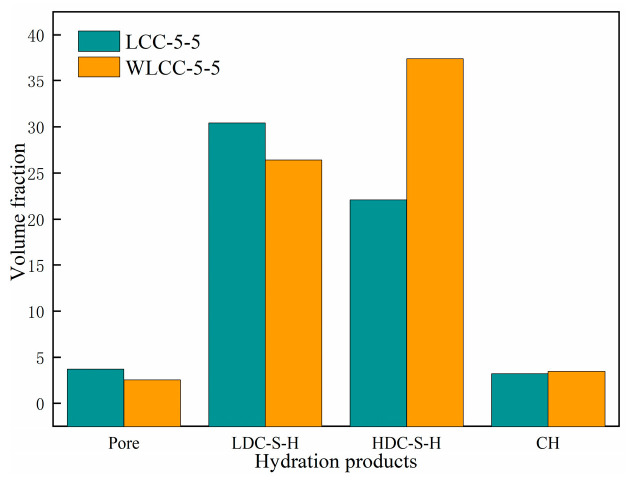
Volume fractions of hydration products in WLCC-5-5 and LCC-5-5.

Simultaneously, with reference to Figure 12, it is observable that the volume fraction of capillary pores in WLCC-5-5 decreases by 27.55% compared to LCC-5-5, consistent with the earlier MIP results. The volume fraction of LDC-S-H gel experiences a reduction of 22.76%. Conversely, the volume fractions of HDC-S-H and CH increased by 13.91% and 23.46%, respectively. This implies that the increased presence of HDC-S-H gel and CH in WLCC-5-5 is advantageous for micro-mechanical properties. Drawing from prior studies [46,47], it is established that HDC-S-H gel and CH typically occupy relatively narrow spaces, whereas LDC-S-H is distributed in relatively wider spaces. The significant volumes of HDC-S-H and CH in the activated low-carbon concrete suggest successful filling of mesopores, resulting in a compressed and narrow transition zone. This is consistent with the findings of [48] that the elevated content of high-density hydrated phases also implies that there are more tightly packed narrow zones in the concrete, and this elucidates the optimization of the pore structure in WLCC-5-5 by the MIP test.

Consequently, the primary mechanism through which NaOH influences the micro-mechanical properties of low-carbon concrete becomes evident. The addition of NaOH results in the production of more hydration phases with a higher hardness and modulus within the low-carbon concrete. This leads to a reduction in the content of detrimental pores, coupled with an enhancement in the hardness and modulus of the pore material. Consequently, the thickness of the interface transition zone decreases, contributing to an overall improvement in micro-mechanical properties.

## 4. Conclusions

This study investigates the impact of NaOH on the micro-mechanical properties of the interface transition zone in low-carbon concrete. The examination includes micro-mechanical properties, pore structure, and hydration products before and after activation. The key findings can be summarized as follows:

(1) In comparison to LCC-5-5, WLCC-5-5, the activated low-carbon concrete, demonstrates significant enhancements in indentation modulus and indentation hardness. The average values for the indentation modulus and hardness in the interface transition zone increase by 19.9% and 25.9%, respectively. Moreover, the thickness of the interface transition zone decreases by 10 µm.
(2) Regarding the pore structure, the MIP test reveals a 1.16% reduction in porosity for WLCC-5-5 compared to LCC-5-5. The volume fraction of harmless pores significantly increases, corresponding to a decrease in harmful pores. The addition of NaOH optimizes the pore structure of low-carbon concrete, leading to an improved porous interface transition zone and enhanced micro-mechanical properties.
(3) Analyzing hydration products through Gaussian fitting of nano-indentation statistical data indicates notable improvements in indentation modulus and hardness for various phases in WLCC-5-5 mortar compared to LCC-5-5. Specifically, the indentation modulus and hardness of the pore phase increase by 30.23% and 43.55%, respectively. The indentation modulus of LDC-S-H increases by 20.13%, and its hardness increases by 19.68%, while HDC-S-H experiences an 18% increase in modulus and a 17.79% increase in hardness. Additionally, the volume fraction of capillary pores and LDC-S-H gel in WLCC-5-5 decreases by 27.55% and 22.76%, respectively, whereas the volume fraction of HDC-S-H and CH increases by 13.91% and 23.46%, respectively.

In terms of hydration products, the addition of NaOH promotes the formation of more high-density hydration products within low-carbon concrete, resulting in increased modulus and hardness of pores. This leads to a reduction in the volume fraction of pores and an improvement in the micro-mechanical property of the overall interface transition zone in low-carbon concrete. The results of this paper provide theoretical references and data support for the study of the micromechanical properties of the interfacial transition zone of composite cementitious materials in the NaOH environment, and also provide a direction for the mixed utilization of glass powder and zeolite powder. However, the comprehensive performance of building materials not only depends on the performance of the interfacial transition zone, but also relates to its overall mechanical properties, work performance, and durability; it is hoped that scholars will conduct in-depth research on these aspects in the future to provide comprehensive support for the application of these materials.

## Figures and Tables

**Table 1:** The main chemical components of ZP and GP.

Chemical Components	SiO_2_ (%)	Al_2_O_3_ (%)	CaO (%)	Fe_2_O_3_ (%)	K_2_O (%)	Na_2_O (%)	C (%)	MgO (%)	Others (%)
ZP	43.5	9.0	4.0	3.1	1.9	2.0	1.8	1.2	2.8
GP	78.3	12.7	1.7	1.5	1.4	2.2	--	1.4	9.5

**Table 2:** Low-carbon concrete mixture ratio.

Sample	Cement	ZP	GP	River Sand	Activator	Coarse Aggregate	Water	Additives
	(kg/m^3^)	(kg/m^3^)	(kg/m^3^)	(kg/m^3^)	(kg/m^3^)	(kg/m^3^)	(kg/m^3^)	(kg/m^3^)
OC	489.8	0.0	0.0	761.9	0	1777.7	210.6	3.3
LCC-4-6	244.4	97.8	146.6	761.9	0	1777.7	210.6	3.3
LCC-5-5	244.4	122.2	122.2	761.9	0	1777.7	210.6	3.3
LCC-6-4	244.4	146.6	97.8	761.9	0	1777.7	210.6	3.3
WLCC-4-6	244.4	97.8	146.6	761.9	9.78	1777.7	210.6	3.3
WLCC-5-5	244.4	122.2	122.2	761.9	9.78	1777.7	210.6	3.3
WLCC-6-4	244.4	146.6	97.8	761.9	9.78	1777.7	210.6	3.3

**Table 3:** Roughness analysis of ITZ samples for each group of concrete.

ITZ Area Sample	Mean Roughness (nm)	Maximum Indentation Depth (nm)
OC	6.74	300
LCC-4-6	5.36	320
LCC-5-5	5.98	310
LCC-6-4	5.25	320
WLCC-4-6	11.66	300
WLCC-5-5	13.78	290
WLCC-6-4	12.25	300

**Table 4:** Average values of nanoindentation modulus and hardness statistical results.

Sample	Aggregate		ITZ		Mortar	
	Modulus	Hardness	Modulus	Hardness	Modulus	Hardness
	GPa	GPa	GPa	GPa	GPa	GPa
OC	82.3509	2.5661	35.2561	1.5798	43.2451	2.8712
LCC-4-6	81.4463	2.4189	24.2109	0.8923	35.2312	1.5045
LCC-5-5	82.8098	3.5013	29.1618	1.2301	38.4731	2.5177
LCC-6-4	83.6028	3.0202	22.0233	0.8201	34.1540	1.4518
WLCC-4-6	80.3681	2.6577	30.4457	1.0132	40.2536	1.8024
WLCC-5-5	83.2445	3.4881	34.9664	1.5496	43.4503	2.7562
WLCC-6-4	81.6743	2.9231	30.0561	0.9926	39.8703	1.7901

**Table 5:** Indentation modulus, hardness, and volume fraction of different hydration products.

Sample	Hydration Products	Modulus		Hardness		Volume Fraction/%
		Mean ± Standard Deviation/GPa	Relative Error/%	Mean ± Standard Deviation/GPa	Relative Error/%	
LCC-5-5	Pore	7 ± 1.90	1.41	0.21 ± 0.17	1.14	3.75
	LDC-S-H	16 ± 2.50	1.70	0.64 ± 0.20	2.16	30.43
	HDC-S-H	33 ± 3.13	0.56	1.20 ± 0.13	1.08	22.10
	CH	39 ± 1.11	2.31	1.59 ± 0.31	0.48	3.24
WLCC-5-5	Pore	10 ± 2.35	2.10	0.373 ± 0.20	1.98	2.51
	LDC-S-H	19 ± 3.13	0.38	0.78 ± 0.17	0.75	26.43
	HDC-S-H	35 ± 2.84	2.06	1.39 ± 0.25	1.39	37.43
	CH	43 ± 1.12	1.31	1.86 ± 0.21	2.55	3.5

## Data Availability

The data utilized in the present work can be obtained from this article.
